# Spatiotemporal Variation of the Association between Urbanicity and Incident Hypertension among Chinese Adults

**DOI:** 10.3390/ijerph17010304

**Published:** 2020-01-01

**Authors:** Jinjing Wu, Jia Chen, Zhen Li, Boshen Jiao, Peter Muennig

**Affiliations:** 1 Asian Demographic Research Institute, Shanghai University, Shanghai 200444, China; jenneyli@163.com; 2 Department of Social Work, Shanghai University, Shanghai 200444, China; chenjia_ling_cool@126.com; 3 The Comparative Health Outcomes, Policy, and Economics (CHOICE) Institute, University of Washington, Seattle, WA 98195, USA; jiaoboshen@gmail.com; 4 Department of Health Policy and Management, Columbia University, New York, NY 10032, USA; pm124@cumc.columbia.edu

**Keywords:** urbanization, urbanicity, incident hypertension, China, adults

## Abstract

Urbanization is believed to result in a transition towards energy-dense diets, sedentary lifestyles, and a subsequent increase in the burden of hypertension (HTN) and other cardiovascular diseases (CVDs) in developing countries. However, the extent to which this occurs is likely dependent on social contexts. We performed multilevel logistic regression models to examine whether the association between incident HTN and the degree to which a community exhibits urban features varied by region (the Northeast, East Coast, Central, and West) within China and period. We used longitudinal data from the China Health and Nutrition Survey (1991–2015) and stratified analyses by sex. Among women, the positive association between medium-to-high urbanicity and HTN onset generally shifted to negative between 1991 and 2015. The high urbanicity was associated with lower odds of developing HTN in the East Coast from the early 1990s. The negative association between high urbanicity and HTN occurrence became statistically significant during 1991–2015 in the Northeastern and Central Regions, while the association remained positive and non-significant in the West. Among men, the relationship between urbanicity and incident HTN was generally non-significant, except for the East Coast in which the negative association between high urbanicity and HTN occurrence became statistically-significant in more recent years. Our findings suggest that, when a subnational region or the society as a whole has become more economically developed, higher urbanicity might turn out to be a protective factor of cardiovascular health. Moreover, improvements made to communities’ urban features might be more effective in preventing HTN for women than for men.

## 1. Introduction

Cardiovascular diseases (CVDs) have become the leading cause of death in both China and the world [1,2]. Hypertension (HTN) is one of the key predictors of cardiovascular mortality and morbidity [3,4]. Although HTN prevalence has been decreasing in the high-income countries since 2000, it has been increasing in low- and middle-income countries, like China (Mills 2016) [5].

There is an increasing consensus regarding the critical role of environments in shaping human behaviors and CVDs, including HTN [6,7,8,9]. China has undergone rapid urbanization over the past four decades, with major consequences for environments and population health. On the one hand, the proportion of population living in urban areas has exceeded 50% since 2011, due to the rural-to-urban migration [10]. On the other hand, many areas that are officially regarded as rural have been gaining urban features [11]. Urban environments were believed to promote lifestyles that place people at risk of HTN [10], as cross-sectional studies in the context of developing countries often showed that urban residents had higher HTN prevalence [12,13]. Hence, urbanization has come to be considered to be one of the forces underlying a nutrition transition towards inactivity coupled with energy-dense diets, and a subsequent increase in the HTN burden [10,14,15]. However, some developed countries may have been experiencing a later stage of nutrition transition involving health-conscious behavioral changes [16]. In those wealthy nations, urbanites tend to eat healthy foods, exercise recreationally [17], and have lower HTN prevalence [18]. When a developing country becomes more economically developed, urban environments may promote healthy behavior changes. Thus, urbanites may take a lead in shifting the population to the transition towards slowing down the growing HTN burden.

China provides a unique opportunity to test this argument. The average living standard has been significantly improved since the establishment of the Reform and Opening-up policy in 1978. However, the improvement in the living standard of rural residents has lagged far behind that of their urban peers. In response, the central government has implemented “urban-rural coordination development” since the early 2000s, which has resulted in significant rural development [19]. Thus, the least and more urbanized populations may have been progressing through the nutrition transition and epidemiological transition at different rates [20], which might have resulted in a changing urban-rural distribution of HTN burden. Additionally, the Northeastern, East Coastal, Central, and West Regions vary substantially with respect to economic development and population health [1,19,21]. The effect of urban environments on HTN might be different among these regions.

Empirical studies in the Chinese context regarding the association between the degree to which a place exhibits urban features and HTN prevalence have provided mixed results [22,23,24,25]. One possible explanation for the inconsistent findings is the secular change in the association, as has been reported in a recent study [21]. Alternatively, the mixed findings may be due to the regional heterogeneity [18]. However, a limitation with existing literature is that the association is often assumed to be uniform in different regions. Exploring subnational regional heterogeneity in the association is critical to a better understanding of the influences of urbanization on the burden of HTN in different social contexts and for developing region-specific strategies for HTN prevention. Moreover, although prevalent HTN is an important measure of HTN burden and it has been widely examined in previous studies [22,23,24,25], it is a less useful measure in etiological studies than incident HTN [26]. Additionally, the urban-rural distribution of HTN and related risk factors seems to be inconsistent in men and women in developing nations [27,28]. Studies that include men and women, but do not analyze data accordingly, may limit the potential for sex-specific findings [29].

With data from the China Health and Nutrition Survey (1991–2015), we aimed to separately test two hypotheses for men and women: (1) the association between urbanicity and incident HTN shifted from positive to negative during 1991–2015 and (2) the association varied by region, with a positive association in the least developed region (the West), and a negative association in the other more developed regions.

## 2. Materials and Methods

### 2.1. Data

We analyzed data from the China Health and Nutrition Survey (CHNS) (1991–2015), a longitudinal study. It covers regions that vary in socioeconomic development, geography, and population health. It was designed to examine the effect of social changes on population health among Chinese.

The first wave was conducted in 1989. Nine subsequent waves were conducted in 1991, 1993, 1997, 2000, 2004, 2006, 2009, 2011, and 2015. The CHNS applied a multistage, random cluster design to draw samples from eight provinces (Liaoning, Henan, Hubei, Hunan, Shandong, Jiangsu, Guizhou, and Guangxi) [30]. Heilongjiang Province was added from 1991, and Beijing, Chongqing, and Shanghai were added from 2011. Further details regarding the CHNS were reported in [30,31].

We excluded the 1989 wave from our analyses, because only people aged 20–45 were sampled in that year. We excluded samples of Beijing, Chongqing, and Shanghai, as one of our objectives is to examine whether the association between the levels of urbanicity and incident HTN changed from 1991 to 2015, because they were only surveyed from 2011.

### 2.2. Sample Selection

In our study, eligible respondents were those who were (1) not pregnant during the physical examination, (2) 20–64 years old, (3) nonhypertensive when they were first surveyed, and (4) had at least one follow-up examination. We dropped subsequent observations after the occurrence of HTN. Therefore, 11,284 respondents, with 52,100 observations, were eligible for our analyses.

**Figure 1:**
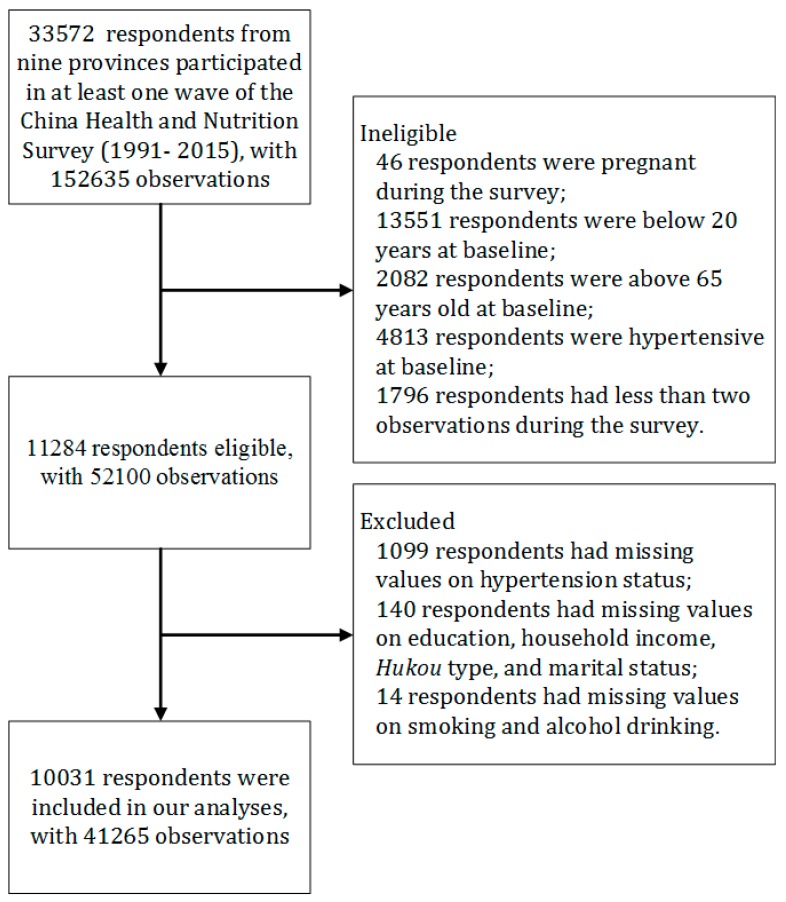
Sample selection process.

We performed complete-case analyses; therefore, we excluded respondents who had missing values on HTN status, educational attainment, household income, *hukou* type, marital status, smoking, and alcohol drinking status, which resulted in an analytic sample of 10,031 respondents, with 41,265 observations. Figure 1 shows the sample selection process. We conducted multiple imputations and rebuilt models based on the multiply imputed datasets in sensitivity analyses while considering the potential bias caused by missing values.

### 2.3. Dependent Variable

The dependent variable was incident HTN (i.e., becoming hypertensive in follow-up examinations or non-hypertensive). Each respondent’s seated systolic/diastolic blood pressure (SBP/DBP) was measured on the right arm while using standard mercury sphygmomanometers by experienced physicians who had attended a seven-day data-collection training session and passed a test for the reliability of blood pressure measurement [32]. SBP and DBP were measured three times after a five-min. rest, and three measurements were taken with a 30-s interval between cuff inflation [32]. We averaged the three readings of SBP and DBP, respectively. The HTN event occurred if respondents had an average SBP higher than 140 mmHg, or an average DBP higher than 90 mmHg, or reported a diagnosis of HTN, or reported taking antihypertensive medicine in follow-up surveys.

### 2.4. Explanatory Variables

#### 2.4.1. Communities’ Urbanicity

We utilized a time-varying scale of urbanicity developed by Jones-Smith and Popkin (2010) to measure the degree to which a community exhibits urban features [33]. The scale is based on both the community-level and individual-level data of CHNS. Jones-Smith and Popkin (2010) identified 12 components that define features of urban places, including population density, traditional markets, modern markets, transportation infrastructure, communications, sanitation, health infrastructure, social services, housing, economic activity, education, and diversity (Table A1 in Appendix A) [33]. Each component was assigned 10 points, equally weighted, and then summed to obtain a total score. The maximum of the total score is 120. A higher urbanicity score indicates that a community is more urbanized. The urbanicity scale has been shown to have very good internal consistency (Cronbach’s alpha = 0.85–0.89) [33].

Instead of categorizing the degree of urbanicity into tertiles [25], we categorized it into quartiles that represent low (urbanicity scores < 57.15), medium-to-high (57.15 ≤ urbanicity scores < 75.09), and high urbanicity (urbanicity scores ≥ 75.09). The low urbanicity was the reference. In our preliminary analyses, we built a model categorizing the degree of urbanicity into quartiles and a model categorizing the degree of urbanicity into tertiles. We found that the former had a better model fit (Table S1 in Supplementary Materials). Additionally, we built a model treating the urbanicity score as continuous and included the score and its quadratic term in our analyses. We still found that the model with medium-to-high urbanicity and high urbanicity had a better fit (Table S2 in Supplementary Materials). Therefore, we treated the degree of urbanicity as a categorical variable and included the medium-to-high urbanicity and high urbanicity in our analyses.

#### 2.4.2. Regions

China can be divided into four economic regions, including the East Coast, Central, West, and Northeast, according to the National Bureau of Statistics of China [34]. Therefore, we grouped our samples into the four regions, which is consistent with previous studies [19,21]. Heilongjiang and Liaoning belong to the Northeast. Shangdong and Jiangsu belong to the East Coast. Henan, Hubei, and Hunan belong to the Central Region. Guizhou and Guangxi belong to the West. The West Region is the reference group.

#### 2.4.3. Survey Years

We used the survey years as a continuous variable and centered it at 1991, with the year 1991 as the reference.

### 2.5. Control Variables

We included demographic variables in our analyses, including sex, age, *hukou* type, and marital status. In terms of age, we calculated how many years had passed, since the respondent’s 20th birthday and included the estimate and its quadratic term in our analyses. We did not include its cubic term, as the model fit had no significant improvement after including the term. *Hukou*, the household registration system in China, categorizes registered residents into agricultural and nonagricultural groups and determines the eligibility for public service and welfare benefits (e.g., education, health care) [35]. As a previous study found that the *hukou* type was associated with prevalent HTN [35], we included it in our analyses to estimate the association between urbanicity and HTN onset, independent of the institutional effect of *hukou*. The CHNS only asked respondents about their *hukou* type from 1993. Therefore, we assumed that respondents’ *hukou* type in 1991 was the same as their *hukou* type in their earliest follow-up surveys. Additionally, we included educational attainment, household income per capita, marital status, smoking, and alcohol drinking status. We did not include diets, physical activity, and body mass index (BMI), because these are intermediate variables between levels of urbanicity and HTN occurrence. Our main objective is to examine whether the levels of urbanicity were associated with HTN occurrence. Including intermediate variables might bias the estimation. Table 1 reports the definitions and distributions of independent and control variables that were included in our analyses. All of the variables included in our analyses are time varying, except for region and sex.

### 2.6. Statistical Analyses

We used a three-level logistic model with two random intercept equations to account for the dependence of residents within communities and repeated observations of individuals to examine the association between communities’ urbanicity and individual-level incident hypertension. Level 1, 2, and 3 were at the observation, individual, and community levels, respectively.

$$y_{\mathrm{ijk}}=\mathrm{logit}\left[p\right]=\log\left[\frac{p}{1-p}\right]=\beta_{0}+\beta_{1}\mathrm{URBAN}+\beta_{2}\mathrm{REGION}+\beta_{3}\mathrm{PERIOD}\;+\beta_{4}\mathrm{URBAN}*\mathrm{REGION}+\beta_{5}\mathrm{URBAN}*\mathrm{PERIOD}+\mu_{j}+\mu_{k}+e_{\mathrm{ijc}}$$

where $y_{\mathrm{ijc}}$ is the log-odds of the *i*th observation of the *j*th person in the *k*th community becoming hypertensive. URBAN, REGION, and PERIOD represent the indicators of urbanicity levels, regions, and survey years, respectively. We added URBAN*REGION, an interaction between levels of urbanicity and regions to examine whether the association between urbanicity and incident HTN varied by region. To examine whether the association changed from 1991 to 2015, we added URBAN*PERIOD, an interaction between levels of urbanicity and survey years. We stratified our analyses by sex.

We used the MELOGIT command in Stata 16.0 (StataCorp, College Station, TX, USA) to perform multilevel logistic regression. We used the LINCOM command and used the MARGINS command in Stata 16.0 to estimate odds ratios and predicted probabilities of developing HTN during the follow-up surveys by urbanicity level, region, and survey year, respectively, to facilitate the interpretation of the interaction effects.

### 2.7. Sensitivity Analyses

We conducted the complete-case analyses in our primary analyses. 1253 respondents (11.10% of eligible respondents) were excluded from the complete-case analyses because of missing data, which may bias the estimates. We compared results from the complete-case analyses with those from multiple imputations to test whether our findings changed when the missingness of data was accounted for. We performed multiple imputations with the MICE (multiple imputation by chained equation) package in R (version 3.6.0, R Software, Miami, FL, USA). Five multiply imputed datasets were generated. We then used the lme4 (linear mixed-effects models) package to build the multilevel logistic regression model on each dataset and pooled estimates of each model into a single set of coefficients and standard errors.

## 3. Results

Among 10,031 respondents, approximately 40.37% developed HTN during the follow-up surveys (male, 46.12%; female, 35.91%). Table 2 reports estimates of multilevel logistic analyses among men and women, respectively. Without including the interactions between urbanicity and regions, or survey years, Model 1 estimated the average effect of urbanicity on HTN occurrence. According to Model 1’s estimates, women who lived in communities with high urbanicity had significantly lower odds of becoming hypertensive than their counterparts living in communities of low urbanicity. However, we found no significant difference in odds of developing HTN between the medium-to-high urbanicity and the low urbanicity. Among men, the medium-to-high and high urbanicity were both not significantly associated with incident HTN. For both sexes, the odds of developing HTN increased across age. Persons who lived in the Northeast, East Coast, and Central had higher odds of becoming hypertensive than their counterparts in the West. Furthermore, we saw an increase in the odds of developing HTN from 1991 to 2015.

We added the interactions between levels of urbanicity and regions in Model 2 to examine the regional variation in the association of levels of urbanicity with HTN occurrence. The model fit improved for both sexes after including the interactions. The coefficients of medium-to-high urbanicity and high urbanicity in Model 2 represent the association between urbanicity and incident HTN in the West. Among women in the West, the medium-to-high urbanicity was significantly positively associated with incident HTN, while the positive association between high urbanicity and HTN onset was positive but non-significant. All of the coefficients of interactions between high urbanicity and regions were negative, and their absolute values were larger than the coefficient of high urbanicity, which indicated that the high urbanicity was inversely related to the odds of developing HTN in the Northeast, East Coast, and Central. The coefficients of interactions between medium-to-high urbanicity and regions were also negative, but only the interaction between medium-to-high urbanicity and the East Coast was statistically significant. Among men, however, none of the interactions between levels of urbanicity and regions were statistically significant.

We added the interactions between urbanicity and survey years in Model 3 to account for the secular change in the association between urbanicity and incident HTN during 1991–2015. The model fit improved for the female samples, but not for the male samples. In Model 3, the coefficients of medium-to-high urbanicity and high urbanicity represent the association between levels of urbanicity and HTN onset in the year 1991 in the West. The interaction between medium-to-high urbanicity and survey years was negative and statistically significant, while the interaction between high urbanicity and survey years was weak and non-significant. However, among males, none of the interactions between the levels of urbanicity and survey years were statistically significant.

**Figure 2:**
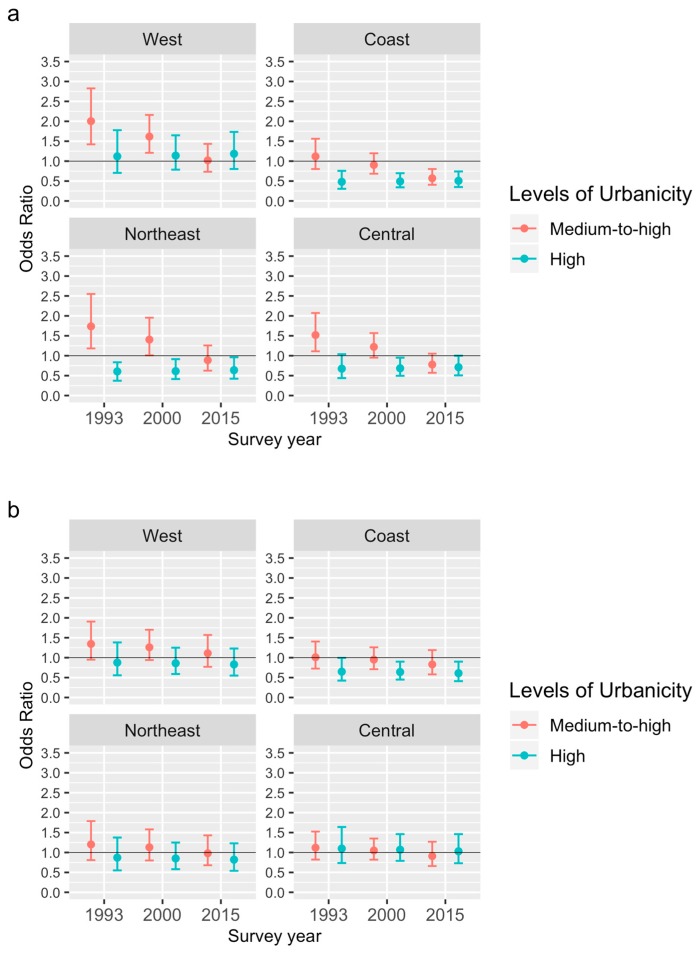
Odds ratio of getting hypertension during the follow-up surveys (the medium-to-high urbanicity, the high urbanicity, versus the low urbanicity) among women (**a**) and men (**b**) by region and survey year.

Based on Model 3’s estimates, Figure 2a,b present the odds ratio of developing HTN living in communities of higher urbanicity versus communities with the low urbanicity level in different survey years in women and men, respectively. Among women in 1993, as shown in Figure 2a, the medium-to-high urbanicity was significantly positively associated with odds of becoming hypertensive, except that the positive association was non-significant in the East Coast. The high urbanicity was inversely related to incident HTN in 1991, except for the West, although only the association in the East Coast was statistically significant. However, the association between high urbanicity and incident HTN was positive but non-significant, among females in the West in 1991.

**Figure A1:**
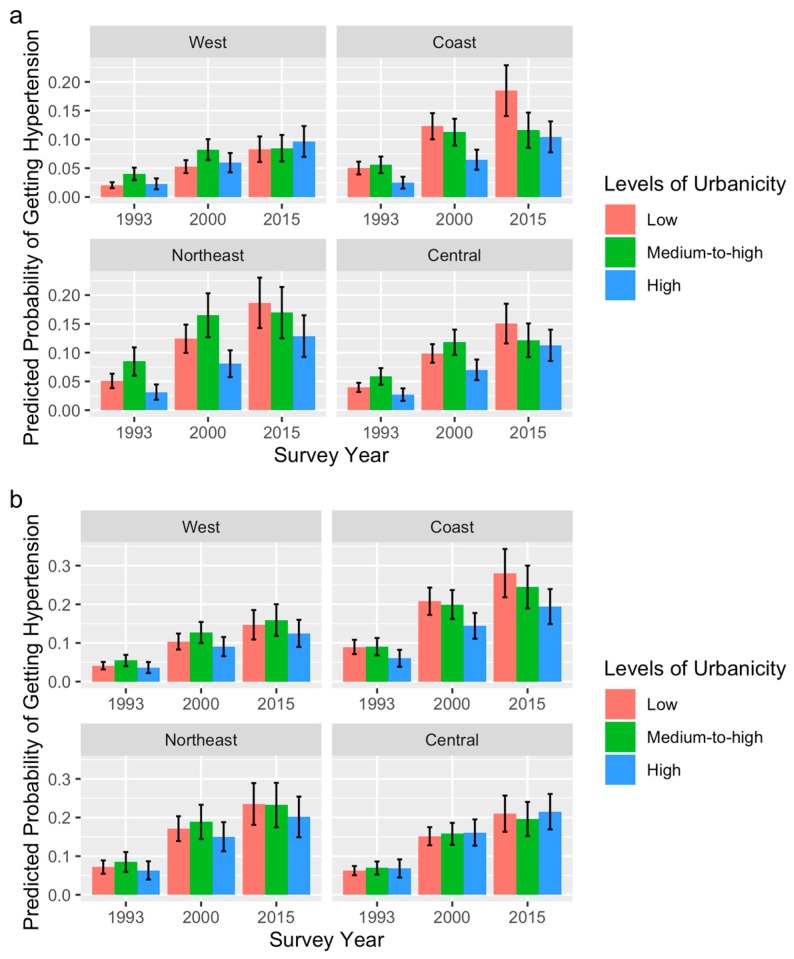
Predicted probabilities of getting hypertension and 95% confidence interval during 1991–2015 among women (**a**) and men (**b**) by levels of urbanicity, region, and survey year.

We also reported the predicted probabilities of developing HTN during the follow-up surveys by urbanicity level, region, and survey year among women and men in Figure A1a,b in Appendix A, respectively. As shown in Figure A1a, there was an inverted U-shaped relationship between the levels of urbanicity and predicted probabilities of developing HTN during the follow-up surveys among women in 1993.

From 1991 to 2015, the magnitude of the positive association of medium-to-high urbanicity with HTN onset among women significantly decreased (Figure 2a). The association turned out to be negative in more recent years, except for the West. The low-urbanized females had a more rapid increase in predicted probabilities of developing HTN relative to their counterparts from communities with the medium-to-high urbanicity, especially during 2000–2015, resulting in the change in the direction of the association of the medium-to-high urbanicity with incident HTN, as shown in Figure A1a in Appendix A.

Furthermore, the negative association between the high urbanicity and incident HTN among females in the Northeastern and Central Regions became statistically significant across the period (Figure 2a). The negative association among the East Coastal females remained statistically significant during the period, while, among their peers in the West, the association was still positive and non-significant in 2015.

Among East Coastal females, the association between levels of urbanicity and predicted probabilities of developing HTN shifted from an inverted U-shaped relationship to a negative gradient from the early 2000s, as shown in Figure A1a in Appendix A. Northeastern and Central females showed a similar trend, although the negative gradient appeared later than their counterparts in the East Coast.

Among men, as shown in Figure 2b, the association between urbanicity and incident HTN was generally non-significant, except for the East Coast. There, the negative association between the high urbanicity and incident HTN became statistically significant during 1991–2015.

In terms of control variables, according to Model 3, among females, having at least upper-secondary education was associated with lower odds of developing HTN. However, household income, nonagricultural *hukou*, being married, current smoking, and alcohol drinking were not significantly associated with incident HTN. Among males, nonagricultural *hukou* and alcohol drinking were significantly associated with higher odds of developing HTN, while household income, educational attainment, married status, and current smoking were not significantly related to incident HTN.

## 4. Discussion

Our study contributes to the ongoing discussion of the role of urbanization in the growing burden of HTN in developing nations by examining the spatiotemporal variation in the association of the degree to which a community exhibits urban features with individual-level incident HTN among Chinese adults. Among women, the association between urbanicity and HTN occurrence varied by region and changed during 1991–2015, which supported our hypotheses and suggested that the association between urbanicity and HTN occurrence was contingent upon social contexts. However, the association between urbanicity and HTN onset among men was generally non-significant, except for the East Coast, which suggested that the role of urbanization in the burden of HTN was also patterned by sex.

A prior study in the Chinese context suggested that living in a more urbanized community was related to a higher risk of having diagnosed chronic diseases, supporting the argument for an urban health penalty [24]. However, our findings suggest that urban residence might not necessarily be related to higher odds of becoming hypertensive during 1991–2015. There are several explanations for the inconsistent results. Firstly, our study focused on HTN, while the prior study included other diagnosed chronic diseases (e.g., diabetes, stroke) [24]. Further studies are needed to examine whether urbanicity is related to the occurrence of other chronic diseases. Secondly, our analyses were mainly based on objectively measured HTN, instead of self-reported diagnosis utilized in the prior study. The higher-urbanized group’s higher likelihood of getting diagnosed than the low-urbanized group might, to some extent, explain the former group’s higher probability of having diagnosed chronic diseases. Thirdly, we focused our analyses on incident HTN during 1991–2015, while they studied prevalent chronic diseases. The higher prevalence of chronic diseases among the more urbanized might be attributed to their survival advantage after obtaining chronic conditions over their counterparts of the least urbanized.

We found that the medium-to-high urbanicity was once positively related to HTN occurrence among women. Consistently, earlier cross-sectional studies suggested that urban residents had higher HTN prevalence than rural residents [12]. However, we could not conclude that the medium-to-high urbanicity was related to worse cardiovascular health, as the positive relationship between the medium-to-high urbanicity and incident HTN in women generally shifted to negative during 1991–2015. Consistently, a prior study reported that rural Chinese had a more apparent increase in HTN incidence than their urban counterparts during 1991–2009 [36]. Zhang (2019) also showed that the association between urbanicity and prevalent HTN shifted from positive to negative during 1991–2011 [25].

However, we further found that the high urbanicity was generally related to lower odds of becoming hypertensive among females, except for the West. Additionally, the secular change in the association of the high urbanicity with HTN occurrence was non-significant for both sexes. Liang et al. (2014) based their analyses on a traditional urban/rural dichotomous variable [36], which might obscure the heterogeneity within the urban/rural administrative areas. Although Zhang (2019) used the urbanicity scale, the association of urbanicity with prevalent HTN was implicitly assumed to be linear [25]. Thus, the different secular trends of the medium-to-high and high urbanicity may have been obscured.

China has achieved rapid economic growth since the economic reform began in the late 1970s. Urban Chinese have obtained more notable increases in income and living standards than their rural peers. It is highly likely that people living in the more urbanized communities have experienced the transition towards high-calorie intake, low energy expenditure, and a subsequent increase in the risk of developing HTN ahead of their peers living in the least urbanized communities. Therefore, it is not surprising to see that the medium-to-high urbanicity was once associated with higher odds of becoming hypertensive among women. However, high urbanicity was associated with lower odds of incident HTN in women from the early 1990s, with the only exception of the West. This finding might seem unexpected given earlier cross-sectional studies showing that urban areas had higher HTN prevalence than rural areas [12]. One possible explanation is that the highly urbanized females in the more developed regions may have experienced the transition towards energy imbalance and increased risk of becoming hypertensive before the 1990s, which is out of our observation period.

The central government has implemented “urban-rural coordination development” to improve rural residents’ living standards since the turn of the new millennium [19]. The least urbanized might have been experiencing the transition towards energy-dense diets and sedentary lifestyles since that time, which results in a catch-up growth in the risk of developing HTN in more recent years. However, among the more urbanized population, there is evidence showing a counter-trend towards high-fat foods and inactivity. For instance, the more urbanized had decreased their intake of animal-source foods since the early 2000s, despite a rapid increase among the low urbanized during the same period [37]. Additionally, the more urbanized increased their leisure physical activity during 1997–2009, while the low urbanized had very limited leisure physical activity and no notable increase during the same period [38]. To summarize, the low-urbanized females may have been experiencing the transition towards high-calorie intake, low energy expenditure, and increased risk of developing HTN, while the more-urbanized females may have been shifting to the stage of health-conscious behavioral change, which might slow down the increase in the risk of developing HTN. Thus, the positive association between the medium-to-high urbanicity and HTN onset shifted to negative in women during 1991–2015.

Moreover, we found regional heterogeneity in the distribution of predicted probabilities of developing HTN by levels of urbanicity among women. In the East Coast, which is the most economically developed area of China, we found a negative gradient since the early 2000s. The Northeastern and Central Regions’ women also showed a similar pattern, although with some delay. However, in the West, which is the least developed region of China, the association of the medium-to-high urbanicity and high urbanicity with HTN occurrence in 2015 was still positive, but non-significant. A cross-country study showed that urban areas had lower HTN prevalence than rural areas in the middle- and high-income countries, while the reverse was true in low-income countries [18]. However, we could not find any comparable studies that have examined the subnational variation in the association of urbanicity with prevalent/incident HTN.

The regional heterogeneity in the association of urbanicity with incident HTN, together with the secular change in the association among women, implied that high urbanicity might turn out to be a protective factor of cardiovascular health when a subnational region or the whole society becomes more economically developed. The impact of globalization varying by region is one possible explanation for the regional variation in the association. The East Coast had higher exposure to the global market than the other regions, especially the West. A prior study found that foreign direct investment, which is an indicator of economic openness, was negatively associated with BMI in urban areas, but positively associated with BMI in rural areas [39], which suggests that globalization might facilitate urban-rural inequalities in cardiovascular health. Dietary quality is one possible mechanism linking globalization and cardiovascular health inequalities. Globalization might facilitate uneven dietary development between the least and more urbanized population by making energy-dense foods more available and accessible to the least urbanized on the one hand, and promoting better diet quality through increasing the dietary diversity among the more urbanized on the other hand [40]. Prior evidence showed that more highly urbanized adults had lower diet quality before 2004, but higher diet quality after because of a more substantial increase in their diet quality over time [41]. Further studies are needed to test what contextual factors and mechanisms can explain the regional variation and secular change in the association of urbanicity with HTN occurrence.

We obtained different findings from men and women, although we did not directly examine sex differences in the association between urbanicity and HTN onset. Among men, the association of urbanicity with odds of developing HTN was generally non-significant except that the negative association between the high urbanicity and HTN onset in the East Coast became significant during 1991–2015. In some other developing countries, like Mozambique, the urban-rural difference in prevalent HTN was significant in women, but not in men [27]. However, according to Zhang (2019), the findings of the association between urbanicity with prevalent HTN were similar for both sexes [25]. One of the reasons for the inconsistent results is that we examined whether urbanicity was related to HTN occurrence, while Zhang (2019) used prevalent HTN as a dependent variable [25]. One possible explanation for our sex-specific findings is that women are more concerned about health risks [42] and they more likely to utilize preventive care than men [43]. Hence, women’s’ cardiovascular health may be more likely to benefit from greater access to health infrastructure and services in highly urbanized communities than men’s. Further studies are needed to confirm whether the association of urbanicity with incident/prevalent HTN differed by sex and to clarify the mechanisms underlying the sex variation.

Several limitations of this study should be taken into account when interpreting our findings. Firstly, CHNS is not nationally representative, although it covers regions that vary in socioeconomic development, geography, and population health. Therefore, our finding, can only be generalized to the adults of the nine provinces included in our study. Secondly, we based our analyses on respondents’ places of residence at the time of the survey. Healthier people are more likely to migrate from less urbanized communities to more urbanized communities when compared with persons with worse cardiovascular health. They are also more likely to move from less economically developed regions to more developed regions. Thus, the association between communities’ urbanicity and incident HTN and the regional differences in the association may be partly due to the selection effect of movement. Thirdly, this is a correlational study. Quasi-experimental studies are needed to evaluate the cause-and-effect relationship between urbanicity and incident HTN.

## 5. Conclusions

This study adds to the evidence showing that there is spatiotemporal variation in the association of the degree to which a community exhibits urban features with HTN occurrence among Chinese adults. It increases our understanding of the dynamic and complex relationship between urbanization, the nutrition transition, and the epidemiological transition in developing countries. Additionally, it improves our understanding of cardiovascular health inequalities between the least and more urbanized population in transitional societies by showing that the HTN occurrence might be increasingly concentrated in the least urbanized population when a subnational region, or the society as a whole, has become more developed. Moreover, improving communities’ urban features might be more beneficial for women’s cardiovascular health than for men.

## Figures and Tables

**Table 1:** Definitions and distributions of independent variables included in our analyses (N = 41,265 observations), China Health and Nutrition Survey (1991–2015).

Variables	Description	Mean (SD)/%
Urbanicity levels	The degree to which a community exhibited urban features, measured by an urbanicity score	58.11 (20.33)
Low urbanicity	Whether communities’ urbanicity scores were below the median (urbanicity scores < 57.15)	51.05%
Medium-to-high urbanicity	Whether communities’ urbanicity scores were in the third quartile (57.15 ≤ urbanicity scores < 75.09)	24.59%
High urbanicity	Whether communities’ urbanicity scores were in the upper quartile (urbanicity scores ≥ 75.09)	24.36%
Regions		
Northeast	Whether a respondent lived in the Northeast	17.24%
East Coast	Whether a respondent lived in the East Coast	22.63%
Central	Whether a respondent lived in the Central	32.99%
West	Whether a respondent lived in the West	27.13%
Male	Whether a respondent was male	42.36%
Age, year	Number of years since 20th birthday	24.36 (12.18)
Period, year	Survey years centered at 1991	10.53 (7.53)
Married	Whether a respondent had a spouse	89.96%
Educational attainment		
≤Primary education	Whether a respondent’s highest educational attainment was primary education or below	45.61%
Lower secondary education	Whether a respondent’s highest educational attainment was lower-secondary education	31.78%
≥Upper secondary education	Whether a respondent’s highest educational attainment was upper-secondary education	22.61%
Household income per capita, yuan	Household income in 10,000 yuan, inflated to 2015 values	0.86 (1.40)
Non-agricultural *hukou*	Whether a respondent held a non-agricultural *hukou*	38.02%
Smoking	Whether a respondent was a current smoker	30.42%
Alcohol drinking	Whether a respondent drank alcohol during the past year	33.28%

**Table 2:** Estimates of multilevel logistic analyses, complete case analyses, China Health and Nutrition Survey (1991–2015) ^a^.

Variables	Female			Male		
	Model 1	Model 2	Model 3	Model 1	Model 2	Model 3
Intercept	−6.39 ****	−6.61 ****	−6.74 ***	−5.39 ***	−5.43 ***	−5.47 ***
	(0.24)	(0.25)	(0.26)	(0.19)	(0.20)	(0.21)
Age	0.11 ***	0.11 ***	0.11 ***	0.06 ***	0.06 ***	0.06 ***
	(0.01)	(0.01)	(0.01)	(0.01)	(0.01)	(0.01)
Age^2^	−0.00 ***	−0.00 ***	−0.001 ***	−0.00 *	−0.00 *	−0.00 *
	(0.00)	(0.00)	(0.00)	(0.00)	(0.00)	(0.00)
Northeast	0.73 ***	0.96 ***	0.95 ***	0.55 ***	0.59 ***	0.59 ***
	(0.11)	(0.15)	(0.15)	(0.11)	(0.15)	(0.15)
East Coast	0.53 ***	0.92 ***	0.93 ***	0.66 ***	0.83 ***	0.84 ***
	(0.11)	(0.15)	(0.15)	(0.11)	(0.15)	(0.15)
Central	0.47 ***	0.67 ***	0.68 ***	0.45 ***	0.44 **	0.44 ***
	(0.10)	(0.13)	(0.13)	(0.10)	(0.13)	(0.13)
Medium-to-high urbanicity	0.07	0.33 *	0.76 ***	0.05	0.20	0.31
	(0.08)	(0.14)	(0.19)	(0.08)	(0.15)	(0.19)
High urbanicty level	−0.37 ***	0.10	0.11	−0.16	−0.18	−0.12
	(0.10)	(0.17)	(0.25)	(0.10)	(0.18)	(0.25)
Period	0.43 ***	0.43 ***	0.45 ***	0.47 ***	0.47 ***	0.47 ***
	(0.03)	(0.03)	(0.03)	(0.03)	(0.03)	(0.03)
Period^2^	−0.04 ***	−0.04 ***	−0.04 ***	−0.04 ***	−0.04 ***	−0.04 ***
	(0.00)	(0.00)	(0.00)	(0.00)	(0.00)	(0.00)
Period^3^	0.00 ***	0.00 ***	0.001 ***	0.00 ***	0.00 ***	0.00 ***
	(0.00)	(0.00)	(0.00)	(0.00)	(0.00)	(0.00)
Medium-to-high × Northeast		−0.18	−0.14		−0.13	−0.11
High × Northeast		(0.21)	(0.21)		(0.22)	(0.22)
		−0.63 **	−0.62 **		−0.01	−0.01
		(0.23)	(0.23)		(0.24)	(0.24)
Medium-to-high × East Coast		−0.55 **	−0.58 **		−0.28	−0.29
High × East Coast		(0.19)	(0.19)		(0.20)	(0.20)
		−0.81 ***	−0.84 ***		−0.29	−0.30
		(0.22)	(0.22)		(0.22)	(0.22)
Medium-to-high × Central		−0.27	−0.28		−0.18	−0.18
		(0.18)	(0.18)		(0.19)	(0.19)
High × Central		−0.47 *	−0.51 *		0.23	0.22
		(0.21)	(0.21)		(0.21)	(0.21)
Medium-to-high × Period			−0.03 ***			−0.01
			(0.01)			(0.01)
High × Period			0.002			−0.00
			(0.01)			(0.01)
Household income per capita	−0.02	−0.02	−0.02	−0.01	−0.01	−0.01
Lower secondary education	(0.02)	(0.02)	(0.02)	(0.02)	(0.02)	(0.02)
	−0.09	−0.09	−0.09	0.02	0.01	0.01
	(0.07)	(0.07)	(0.07)	(0.07)	(0.07)	(0.07)
Upper secondary education	−0.22 *	−0.22 *	−0.22 *	0.01	0.00	0.00
	(0.09)	(0.09)	(0.09)	(0.08)	(0.08)	(0.08)
Non-agricultural *hukou*	0.03	0.05	−0.02	0.17 *	0.19 *	0.17 *
	(0.08)	(0.08)	(0.08)	(0.08)	(0.08)	(0.08)
Married	−0.09	−0.09	−0.09	−0.07	−0.07	−0.07
	(0.08)	(0.08)	(0.08)	(0.09)	(0.09)	(0.09)
Current smoker	−0.21	−0.22	−0.22	0.02	0.02	0.02
	(0.12)	(0.12)	(0.12)	(0.05)	(0.05)	(0.05)
Drinking alcohol	0.01	0.02	0.01	0.14 **	0.14 **	0.14 **
	(0.09)	(0.09)	(0.09)	(0.05)	(0.05)	(0.05)
Random effects	S.D.	S.D.	S.D.	S.D.	S.D.	S.D.
Community level: intercept	0.14 ***	0.13 ***	0.12 ***	0.14 ***	0.14 ***	0.14 ***
	(0.03)	(0.03)	(0.03)	(0.03)	(0.03)	(0.03)
Individual level: intercept	0.07	0.07	0.04	0.00	0.00	0.00
	(0.13)	(0.12)	(0.12)	(0.00)	(0.00)	(0.00)
AIC	12237.97	12232.66	12222.98	11485.42	11487.49	11490.54
−2 Log Likelihood	−6098.99	−6090.33	−6083.49	−5723.71	−5718.74	−5718.27
Number of observations	23,787	23,787	23,787	17,478	17,478	17,478

* *p* < 0.05, ** *p* < 0.01, *** *p* < 0.001. AIC, Aikake Information Criterion. ^a^ In sensitivity analyses, we rebuilt models based on multiply imputed datasets. We presented estimates in Table S3 of Supplementary Materials. We found that the findings of the association between urbanicity and incident HTN remained unchanged after multiple imputations, indicating that our main results were robust.

## Supplementary Materials

The following are available online at https://www.mdpi.com/1660-4601/17/1/304/s1, Table S1: Estimates of multilevel logistic analyses, comparing a model categorizing the degree of urbanicity into tertiles with a model categorizing the degree of urbanicity into quartiles, China Health and Nutrition Survey (1991–2015), Table S2: Estimates of multilevel logistic analyses, comparing a model treating the degree of urbanicity as continuous with a model treating the degree of urbanicity as categorical, China Health and Nutrition Survey (1991–2015), Table S3: Estimates of multilevel logistic analyses, based on multiply imputed datasets, China Health and Nutrition Survey (1991–2015).

## Appendix A

**Table A1:** Description of the twelve components of the urbanicity scale, China Health and Nutrition Survey (1991–2015).

Components	Variables Used to Define Twelve Components of Urbanicity ^a^	Mean (SD)
Population density	Total population of the community divided by community area	5.82 (1.40)
Traditional markets	Distance to nine types of markets (grains, oil, meat, vegetables, fish, bean curd, milk, fabric and fuel); number of days markets are open	4.97 (3.53)
Modern markets	Number of supermarkets, cafes, internet cafes, indoor restaurants, outdoor fixed and mobile eateries, bakeries, ice cream parlor, fast food restaurant, fruit and vegetable stands and bars within the community boundaries	4.40 (3.11)
Transportation infrastructure	Type of road (dirt/stone/gravel/mixed/paved); distance to bus stop; distance to train stop	5.40 (2.56)
Communications	Availability of a cinema, newspaper, postal service, telephone service; percent of households with a computer; percent of households with a television; percent of households with a cell phone	5.06 (1.73)
Sanitation	Proportion of households with treated water; prevalence of households without excreta present outside the home	6.04 (3.16)
Health infrastructure	Type of health facilities; number of health facilities in or nearby (≤12 km) the community; number of pharmacies in community	5.53 (2.34)
Social services	Availability of a child care center; availability of commercial medical insurance; availability of free medical insurance; availability of insurance for women and children	1.89 (2.61)
Housing	Average number of days a week that electricity is available to the community; percent of community with indoor tap water; percent of community with flush toilets; percent of community that cooks with gas	5.95 (2.84)
Economic activity	Typical daily wage for ordinary male worker; percent of the population engaged in nonagricultural work	4.90 (3.32)
Education	Average education level among adults (>21 years old)	3.24 (1.51)
Diversity	Variation in community education level; variation in community income level	4.63 (1.29)

^a^ The description of variables used to define urbanicity was adapted from Jones-Smith and Popkin (2010) [33], with permission of the publisher. Copyright © Elsevier, 2010.
