# Supplementary material for: Spatiotemporal Variation of the Association between Urbanicity and Incident Hypertension among Chinese Adults
Source: Int J Environ Res Public Health. 2020 Jan 1;17(1):304. doi: 10.3390/ijerph17010304 (PMC6982103; doi:10.3390/ijerph17010304)
Supplement: Supplementary file 1 [file ijerph-17-00304-s001.pdf]

Supplementary Materials

In our preliminary analyses, we compared model A categorizing the degree of urbanicity into quartiles with Model B categorizing the degree of urbanicity into tertiles. Table S1 presents Model A and B's estimates, respectively. By comparing their values of Aikake Information Criterion (AIC), we evaluated if Model A fits the data better than Model B. Model A had a lower AIC value than Model B, suggesting that Model A fits the data better.

Table 1. Estimates of multilevel logistic analyses, comparing Model A categorizing the degree of urbanicity into quartiles with Model B categorizing the degree of urbanicity into tertiles, China Health and Nutrition Survey (1991–2015).

Variables	Female		Male	
	**Model A**	**Model B**	**Model A**	**Model B**
Intercept	−6.39 ***<br>(0.24)	−6.34 ***<br>(0.24)	−5.39 ***<br>(0.19)	−5.35 ***<br>(0.19)
Duration	0.11 ***<br>(0.01)	0.11 ***<br>(0.01)	0.06 ***<br>(0.01)	0.06 ***<br>(0.01)
Duration^2	−0.00 ***<br>(0.00)	−0.00 ***<br>(0.00)	−0.00 *<br>(0.00)	−0.00 *<br>(0.00)
Coastal East	0.53 ***<br>(0.11)	0.52 ***<br>(0.10)	0.66 ***<br>(0.11)	0.65 ***<br>(0.11)
Northeast	0.73 ***<br>(0.11)	0.73 ***<br>(0.11)	0.55 ***<br>(0.11)	0.55 ***<br>(0.11)
Central	0.47 ***<br>(0.10)	0.48 ***<br>(0.10)	0.45 ***<br>(0.10)	0.45 ***<br>(0.10)
Medium-to-high urbanicity<br>(57.15 ≤ urbanicity scores < 75.09)	0.07<br>(0.08)		0.05<br>(0.08)	
High urbanicity<br>(urbanicity scores ≥75.09)	−0.37 ***<br>(0.10)		−0.16<br>(0.10)	
Middle urbanicity level (47.12 ≤ urbanicity scores < 68.71)		0.22 **<br>(0.07)		−0.01<br>(0.08)
High urbanicity<br>(urbanicity scores ≥68.71)		0.07<br>(0.10)		−0.08<br>(0.10)
Period	0.43 ***<br>(0.03)	0.42 ***<br>(0.03)	0.47 ***<br>(0.03)	0.46 ***<br>(0.03)
Period^2	−0.04 ***<br>(0.00)	−0.04 ***<br>(0.00)	−0.04 ***<br>(0.00)	−0.04 ***<br>(0.00)
Period^3	0.00 ***<br>(0.00)	0.00 ***<br>(0.00)	0.00 ***<br>(0.00)	0.00 ***<br>(0.00)
Household income per capita	−0.02<br>(0.02)	−0.03<br>(0.02)	−0.01<br>(0.02)	−0.02<br>(0.02)
Lower secondary education	−0.09<br>(0.07)	−0.11<br>(0.07)	0.02<br>(0.07)	0.02<br>(0.07)
Upper secondary education	−0.22 *<br>(0.09)	−0.27 **<br>(0.09)	0.01<br>(0.08)	0.00<br>(0.08)
Non-agricultural *Hukou*	0.03<br>(0.08)	−0.11<br>(0.08)	0.17 *<br>(0.08)	0.15<br>(0.08)
Married	−0.09<br>(0.08)	−0.10<br>(0.08)	−0.07<br>(0.09)	−0.07<br>(0.09)
Current smoker	−0.21<br>(0.12)	−0.20<br>(0.12)	0.02<br>(0.05)	0.02<br>(0.05)
Drinking alcohol	0.01<br>(0.09)	0.01<br>(0.09)	0.14 **<br>(0.05)	0.14 **<br>(0.05)
Random effects	S.D.	S.D.	S.D.	S.D.
Community level: intercept	0.14 ***<br>(0.03)	0.13 ***<br>(0.03)	0.14 ***<br>(0.03)	0.14 ***<br>(0.03)

Individual level: intercept	0.07<br>(0.13)	0.03<br>(0.12)	0.00<br>(0.00)	0.00<br>(0.00)
AIC	12,237.97	12,253.36	11,485.42	11,491.41
−2 Log Likelihood	−6098.99	−6106.68	−5723.71	−5726.71
Number of observations	23,787	23,787	17,478	17,478

* p < 0.05, ** p < 0.01, *** p < 0.001. AIC, Aikake Information Criterion.

In our preliminary analyses, we built Model A treating the degree of urbanicity as categorical and Model B treating the degree of urbanicity as continuous. Table S2 presents Model A and B's estimates, respectively. By comparing their AIC values, we found Model A had a lower AIC value than Model B, suggesting that Model A fits the data better. Therefore, we treated the degree of urbanicity as categorical rather than continuous in our final analyses.

Table 2. Estimates of multilevel logistic analyses, comparing Model A treating the degree of urbanicity as categorical with Model B treating the degree of urbanicity as continuous, China Health and Nutrition Survey (1991–2015).

**Variables**	**Female**		**Male**	
	**Model A**	**Model B**	**Model A**	**Model B**
Intercept	−6.39 ***<br>(0.24)	−7.13 ***<br>(0.36)	−5.39 ***<br>(0.19)	−5.58 ***<br>(0.31)
Duration	0.11 ***<br>(0.01)	0.11 ***<br>(0.01)	0.06 ***<br>(0.01)	0.06 ***<br>(0.01)
Duration^2	−0.00 ***<br>(0.00)	−0.00 ***<br>(0.00)	−0.00 *<br>(0.00)	−0.00 *<br>(0.00)
Coastal East	0.53 ***<br>(0.11)	0.53 ***<br>(0.11)	0.66 ***<br>(0.11)	0.66 ***<br>(0.11)
Northeast	0.73 ***<br>(0.11)	0.73 ***<br>(0.11)	0.55 ***<br>(0.11)	0.55 ***<br>(0.11)
Central	0.47 ***<br>(0.10)	0.49 ***<br>(0.10)	0.45 ***<br>(0.10)	0.45 ***<br>(0.10)
Medium-to-high urbanicity<br>(57.15 ≤ urbanicity scores < 75.09)	0.07<br>(0.08)		0.05<br>(0.08)	
High urbanicity<br>(urbanicity scores ≥75.09)	−0.37 ***<br>(0.10)		−0.16<br>(0.10)	
Urbanicity score		0.03***<br>(0.01)		0.01<br>(0.01)
Urbanicity score^2		−0.00***<br>(0.00)		−0.00<br>(0.00)
Period	0.43 ***<br>(0.03)	0.41 ***<br>(0.03)	0.47 ***<br>(0.03)	0.46 ***<br>(0.03)
Period^2	−0.04 ***<br>(0.00)	−0.04 ***<br>(0.00)	−0.04 ***<br>(0.00)	−0.04 ***<br>(0.00)
Period^3	0.00 ***<br>(0.00)	0.00 ***<br>(0.00)	0.00 ***<br>(0.00)	0.00 ***<br>(0.00)
Household income per capita	−0.02<br>(0.02)	−0.02<br>(0.02)	−0.01<br>(0.02)	−0.01<br>(0.02)
Lower secondary education	−0.09<br>(0.07)	−0.10<br>(0.07)	0.02<br>(0.07)	0.01<br>(0.07)
Upper secondary education	−0.22 *<br>(0.09)	−0.25 **<br>(0.09)	0.01<br>(0.08)	0.00<br>(0.08)
Non-agricultural *Hukou*	0.03<br>(0.08)	-0.06<br>(0.09)	0.17*<br>(0.08)	0.16<br>(0.08)
Married	−0.09<br>(0.08)	−0.09<br>(0.08)	−0.07<br>(0.09)	−0.07<br>(0.09)
Current smoker	−0.21<br>(0.12)	−0.20<br>(0.12)	0.02<br>(0.05)	0.03<br>(0.05)

Drinking alcohol	0.01<br>(0.09)	0.01<br>(0.09)	0.14 **<br>(0.05)	0.14 **<br>(0.05)
Random effects	S.D.	S.D.	S.D.	S.D.
Community level: intercept	0.14 ***<br>(0.03)	0.13 ***<br>(0.03)	0.14 ***<br>(0.03)	0.14 ***<br>(0.03)
Individual level: intercept	0.07<br>(0.13)	0.05<br>(0.12)	0.00<br>(0.00)	0.00<br>(0.00)
AIC	12,237.97	12,250.28	11,485.42	11,489.36
−2 Log Likelihood	−6098.99	−6105.14	−5723.71	−5725.68
Number of observations	23,787	23,787	17,478	17,478

* $p < 0.05$, ** $p < 0.01$, *** $p < 0.001$. AIC, Aikake Information Criterion.

In our sensitivity analyses, we performed multiple imputations and created five multiply imputed datasets to examine whether our findings changed after accounting for the missing data. We conducted multilevel logistic regression analyses based on each imputed dataset and then pooled estimates obtained from each dataset. Table S3 reports the pooled coefficients and standard errors. Table S3 shows that the findings of the association between urbanicity levels and HTN occurrence remained unchanged, indicating that our main results were robust. However, the negative coefficient of being married became statistically significant among women, and the positive coefficient of the nonagricultural *Hukou* type became non-significant among men after conducting the multiple imputations.

Table 3. Estimates of multilevel logistic analyses, based on multiply imputed datasets, China Health and Nutrition Survey (1991–2015).

Variables	Female	Male
	**Model 3**	**Model 3**
Intercept	−6.63 ***<br>(0.20)	−5.37 ***<br>(0.18)
Duration	0.11 ***<br>(0.01)	0.07 ***<br>(0.01)
Duration^2	−0.001 ***<br>(0.00)	−0.0004 *<br>(0.00)
Northeast	0.89 ***<br>(0.15)	0.62 ***<br>(0.15)
Coastal East	0.86 ***<br>(0.14)	0.86 ***<br>(0.14)
Central	0.60 ***<br>(0.14)	0.46 ***<br>(0.13)
Medium-to-high urbanicity	0.69 ***<br>(0.19)	0.32<br>(0.19)
High urbanicity	0.07<br>(0.27)	−0.09<br>(0.24)
Period	0.42 ***<br>(0.03)	0.45 ***<br>(0.02)
Period^2	−0.03 ***<br>(0.003)	−0.04 ***<br>(0.001)
Period^3	0.001 ***<br>(0.00)	0.001 ***<br>(0.00)
Medium-to-high × Northeast	−0.04<br>(0.22)	−0.07<br>(0.22)
High × Northeast	−0.54 *<br>(0.25)	-0.10<br>(0.24)
Medium-to-high × Coastal East	−0.43 *<br>(0.20)	−0.32<br>(0.19)
High × Coastal East	−0.77 ***<br>(0.21)	−0.34<br>(0.24)

Medium-to-high × Central	−0.16<br>(0.19)	−0.26<br>(0.17)
High × Central	−0.43 *<br>(0.20)	0.09<br>(0.21)
Medium-to-high × Period	−0.03 **<br>(0.01)	−0.01<br>(0.01)
High × Period	0.003<br>(0.01)	0.002<br>(0.01)
Household income per capita	−0.01<br>(0.02)	−0.02<br>(0.02)
Lower secondary education	−0.07<br>(0.06)	0.005<br>(0.07)
Upper secondary education	−0.23 *<br>(0.09)	−0.03<br>(0.08)
Non-agricultural *Hukou*	−0.06<br>(0.10)	0.13<br>(0.08)
Married	−0.20<br>(0.07) **	−0.09<br>(0.09)
Current smoker	−0.20<br>(0.12)	0.03<br>(0.05)
Drinking alcohol	−0.01<br>(0.10)	0.11 *<br>(0.06)
Number of observations	30,004	22,096

* $p < 0.05$, ** $p < 0.01$, *** $p < 0.001$.
